# It’s all not negative: a cross-section study on the impacts of Covid-19 pandemic on Iranian population

**DOI:** 10.1186/s12889-022-14777-3

**Published:** 2022-12-08

**Authors:** Hossein Namdar Areshtanab, Mina Hosseinzadeh, Maryam Vahidi, Sheila K. Hurst, Soraya Soheili

**Affiliations:** 1 Social Determinants of Health Research Center, Tabriz University of Medical Sciences, Tabriz, Iran; 2 Department of Community Health Nursing, Nursing and Midwifery faculty, Tabriz University of Medical Sciences, Tabriz, Iran; 3 Department of Mental Health and Psychiatric Nursing, Nursing and Midwifery Faculty, Tabriz University of Medical Sciences, Tabriz, Iran; 4 Washington State University, Spokane, Washington USA; 5 Department of Nursing, Marand Branch, Islamic Azad University, Marand, Iran

**Keywords:** Covid-19, Pandemic, Lifestyle, Iran

## Abstract

**Background:**

Covid-19 has had significant effects on the quality of life of individuals around the world. Research highlights many negative impacts related to Covid-19; however, there are also potential positive impacts. The current study aimed to identify both the perceived negative and positive effects of Covid-19 among the residents of Tabriz, Iran.

**Materials & methods:**

The descriptive cross-sectional study was conducted in 10 health centers in Tabriz city in 2021. Cluster random sampling was used to select the 861 study participants. A questionnaire was developed to collect demographic and Covid-19 impacts. Data were analyzed with descriptive and inferential statistics using SPSS 16 software.

**Results:**

The mean (SD) of negative and positive impacts of Covid-19 was 37.7 (5.10) and 36.35 (5.31), respectively. Significant negative impacts included restriction in participation in cultural (90.4%) and religious (88.9%) activities. Positive impacts were increased attention to personal hygiene (88.7%) and attention to elders (87.6%). Gender (*p* = 0.006), work status (*p* = 0.004) and age (*p* = 0. 01) had significant association with the mean of negative impacts and work status (*p* = 0.01), age (*p* < 0.001), history of Covid-19 (*p* = 0.01) and family history of Covid-19 (*p* < 0.001) had the significant association with the mean of positive impacts of Covid-19.

**Conclusion:**

The findings revealed that the general population experienced many negative impacts of Covid-19. This may be related to the priority of religious gatherings in Iranian culture. Despite the unfavorable effects of Covid-19, its positive effects and post-traumatic growth should not be ignored. Covid-19 may be used as an opportunity to develop personal growth and a positive outlook on life. Healthcare workers should reinforce the positive impacts of Covid-19 while taking into consideration the importance of spirituality among Iranian individuals during times of community lockdowns.

## Introduction

Covid-19 is a contagious respiratory disease first identified and confirmed in Wuhan, China [1]. With the rapid and severe increase in cases reported worldwide, the World Health Organization (WHO) declared the outbreak a global pandemic and called on all countries to work together to confront the disease [2]. Until 17 December 2021, all over the world, about 271,963,258 confirmed cases and nearly 5,331,019 deaths have been reported to WHO [3]. WHO reported 16,970,719 confirmed cases in the eastern Mediterranean region and 312,896 deaths. In Iran, from 3 to 2020 to 17 December 2021, there have been 6,160,303 confirmed cases of Covid-19 with 130,831 deaths [4].

Iranian health system aimed to mitigate the spread of Covid-19 through strategies such as lockdowns, social distancing, vaccinations, high protection requirements, and online education instead of face-to-face education [5]. For those who contracted the illness, Iranian health services provided outpatient and inpatient hospital care [6]. However, barriers related to Iranian political and economic sanctions were prevalent. The restriction placed on Iran’s banking system limited the purchasing of essential raw materials for medication synthesis, medical equipment, and supplies necessary for hospitals and health centers to provide adequate care. Essential diagnostic testing equipment (e.g., real-time PCR and X-Ray Machines) and specialized hospital beds were severely limited for critical and intensive care units [7]. These sanctions and lockdown interventions resulted in significant lifestyle changes contributing to health problems such as psychological distress and fear [6].

Studies have demonstrated that following the outbreak of Covid-19, psychological effects such as depression, anxiety, fear, and panic have increased considerably among the general population [8, 9]. Mortality, rapid transmission, prolonged incubation period, intrinsic viral secrecy of unknown origin, imposed lockdown, lack of sufficient knowledge about this disease, and fear of death and pain can also be caused by a phenomenon known as corona-phobia [10, 11].

Not all responses to the challenges posed by Covid-19 were negative. Covid-19 also presented opportunities for positive changes in our life, which were impossible before Covid-19 [12]. The literature demonstrates that traumatic events can broaden one’s attitude, enhance their capacity to manage problems, and increase individual and social skills [13, 14]. In a recent study, 10% of employees perceived a positive impact of the crisis on their work life, and 13% of employees perceived a positive effect on their private life [15].

In Iran, like many other countries, during 2021 and 2022, the authorities have imposed periods of general lockdown to combat coronavirus. The lockdown included the closure of all markets, public offices, schools, universities, movie theaters, and restaurants. Moreover, travel was banned, religious centers were closed, and Friday prayer sessions were suspended. Approximately 99.4% of Iranians are Muslim, and such restrictions hinder their ability to perform their religious duties [5].

Current research highlights psychological and health impacts among individuals around the world [16–18]; however, it is unclear how these impacts differed among the residents of Iran, so we aimed to determine the negative and positive impacts of Covid-19 among the general Iranian population. The findings of this study can potentially guide health policymakers in reducing negative impacts and enhancing positive outcomes related to the pandemic. Further, these findings provide insight into Iranian culture and aid in prioritizing activities to maintain and support quality of life during isolation.

## Methods

In this descriptive cross-sectional study, 861 clients referred to health centers in Tabriz city in 2021 participated. Tabriz city is the most significant economic hub and metropolitan area in the northwest of Iran, with a population of more than 1.6 million. The majority of its people follow the Islamic beliefs of Shia Islam. Simple random sampling was used to select ten health centers (of 83) in Tabriz city. Health centers included vaccination units, physicians, and family health units. Random cluster sampling was then used to select 861 participants. The final sample size was determined using the z2× p(1-p)/d2 formula [with estimated *p* = 0.5, d = 5%, 95% confidence level, and considering design effect 2 and nonresponse rate of 10%. Inclusion criteria included individuals > 18 years old, the ability to understand the Persian language, and willingness to participate in the study. Exclusion criteria were a self-report of having a cognitive or mental disorder and an inability to understand the Persian language. To prevent the transmission of Coronavirus infection, the primary researcher read the questionnaire to participants and recorded the results on a laptop device. An electronic version of the questionnaire was used, and participants completed all questionnaires in a private room to protect the privacy of responses.

Data were collected from August to October 2021 using a face-to-face interview to deliver a 2-part questionnaire. The questionnaire included socio-demographic characteristics and viewpoints about Coronavirus impacts. The questionnaire was designed through a literature review and validated by researchers [19–22]. Socio-demographic characteristics included participants’ age, gender, education level, marital status, number of living children, smoking status, job, history of Covid-19, family history of Covid-19, and sources of information. The questionnaire on Covid-19 impacts included ten statements, each related to positive and negative effects. Each statement was designed based on a 5-point Likert scale (strongly agree, agree, neutral, disagree, strongly disagree), scoring from 1 to 5. The total score range was 10 to 50 for each positive or negative impact.

Both quantitative and qualitative methods determined the face and content validities of the questionnaires. For this purpose, the questionnaires were piloted among ten professors at Tabriz University of Medical Sciences, and based on their feedback, and corrections were made. Content validity for the questionnaire was determined using the criteria by Lawshe. According to the Lawshe table, the content validity ratio (CVR) above 0.62 and content validity index (CVI) above 0.79 is acceptable [23]. In the present study, the ten expert’s views were considered, the CVR for the questionnaire was 0.83, and CVI was 0.90. Exploratory factor analysis (EFA) was used to assess the construct validity using Bartlett’s test (of Homogeneity of Variances), the Kaiser-Meyer-Olkin (KMO) index, screen plots, and Oblimin rotation were used in explanatory factor analysis. The KMO index was 0.84, and Bartlett’s test was 10718.32 at the significance level of (*P* < 0 0.001). Factor loadings obtained for all items in the questionnaire were more than 0.3. Internal consistency reliability was used to determine the reliability of the tool. Cronbach’s alpha coefficient generated 0.89.

### Data analysis

Data were analyzed using SPSS 16. The normality of data was assessed using Skewness and Kurtosis, which had a normal distribution. The Pearson correlation, Independent sample t-test, and one-way analysis of variance were applied to determine the association of positive and negative outcomes with socio-demographic characteristics. Significance was set for *p* < 0.05.

### Ethical consideration

The research proposal was approved by the Ethics Committee of IRB Medical Sciences (ethical code: IR.TBZMED.REC 1399.985). Participants were informed regarding the research goals, anonymity, and voluntary participation, followed by a written and signed informed consent form.

## Results

In this study, 604 (70.2%) participants were female, and 581(67.5%) were single. The demographic characteristics of the participants are shown in Table 1.

**Table 1:** Participants’ demographic characteristics (*n* = 861)

Variable			N (%)	Variable		N (%)
Gender		male	257(29.8)	Education level	High school or less	531(61.7)
		female	604(70.2)		College degree	330(28.3)
Age		18–29	258(30)	History of Covid-19	Yes	313(36.4)
		30–39	180(20.9)			
		40–49	150(17.4)		No	548(63.6)
		50–64	139(18.1)	Marital status	married	264(30.7)
		≥ 65	134(15.6)		single	581(67.5)
					divorced	16(1.9)
Work status		employee	451(52.38)	Family history of Covid-19	yes	527(61.2)
		un employee	155(18)		no	334(38.8)
		Retiree	49(5.69)	Number of children	0–1	422(49)
		Academic student	75(8.71)		2–3	334(38.8)
		housework	131(15.21)		3–4	73(8.5)
Information sources (By priority)	Radio and television	First	648(75.3)		> 5	32(3.7)
		Second	88(10.2)	physicians and nurses	First	31(3.6)
		Third	33(3.8)		Second	30(3.5)
		Fourth	28(3.3)		Third	34(3.9)
		Fifth	8(0.9)		Fourth	108(12.5)
	Newspapers	First	1(0.1)		Fifth	602(69.9)
		Second	135(15.7)	Friends and acquaintances	First	20(2.3)
		Third	460(53.4)		Second	24(2.8)
		Fourth	41(4.8)		Third	124(14.4)
		Fifth	167(19.4)		Fourth	615(71.4)
	Social networks and the Internet	First	105(12.2)		Fifth	22(2.6)
		Second	528(61.3)			
		Third	154(17.9)			
		Fourth	13(1.5)			
		Fifth	6(0.7)			

The mean (SD) of negative impacts (items 1–10) of Covid-19 was 37.7 (5.10). Significant items where participants selected “agree” and “strongly agree” include lack of visitation to cultural centers (90.4%) and inability to attend religious activities (88.9%). The mean (SD) of positive impacts of Covid-19 was 36.35 (5.31). Notable items related to positive effects (items 11 to 20, Table 2) included improved personal hygiene (88.7%) and improved attention to older adults (87.6%). The frequency and percentage of each item related to positive and negative impacts are given in Table 2.

**Table 2:** Frequency and percentage of the statements regarding the impacts of Covid-19 (*N* = 861)

	Statement	Strongly disagree	disagree	Neutral	agree	Strongly agree
		N(% )	N(% )	N(% )	N(% )	N(% )
1	Covid-19 outbreak has led me to restrict travel with relatives.	3(4)	16(1.9)	91(10.7)	486(57.4)	252(29.7)
2	The prevalence of Covid-19 has made it impossible for me to go to cultural centers such as cinemas, theaters and exhibitions.	1(0.1)	15(1.8)	65(7.7)	459(54.3)	305(36.1)
3	The prevalence of Covid-19 has made it impossible for me to go to religious places and gatherings such as mosques and religious congregations.	5(0.6)	23(2.7)	66(7.8)	396(46.8)	357(42.1)
4	Covid-19 outbreak has led me to buy and maintain more of my family’s necessities, such as food and detergent.	25(3)	237(28)	96(11.3)	331(39.1)	158(18.7)
5	Covid-19 outbreak has made it impossible for me to travel as I did in previous years.	4(0.5)	54(6.3)	59(6.9)	478(56.2)	256(30.1)
6	The prevalence of Covid-19 has increased family and marital disputes in our family.	73(8.7)	303(35.9)	103(12.2)	276(32.7)	88(10.4)
7	Covid-19 outbreak has caused me to have more and more psychological symptoms such as fear, anxiety, and guilt and so on.	28(3.3)	322(38.2)	105(12.5)	301(35.7)	87(10.3)
8	The outbreak of Covid-19 has prevented the mourning ceremony for the lost loved ones from being held in full.	4(0.5)	54(6.4)	67(7.9)	373(43.9)	351(41.3)
9	The outbreak of Covid-19 has increased my dependence on cyberspace.	47(5.6)	145(17.2)	87(10.3)	396(47.1)	166(19.7)
10	The outbreak of Covid-19 has caused me to become involved in learning virtually.	34(4.3)	103(12.9)	151(18.9)	335(42)	175(21.9)
11	The outbreak of Covid-19 has caused me to face less traffic load when traveling in the city.	50(5.9)	315(37.3)	143(16.9)	270(32)	66(7.8)
12	The outbreak of Covid-19 has given me ample opportunity to read my favorite books.	46(5.5)	99(11.8)	136(16.2)	454(53.9)	107(12.7)
13	The outbreak of Covid-19 has made me spend more time with my family members.	11(1.3)	62(7.3)	87(10.3)	545(64.3)	143(16.9)
14	The outbreak of Covid-19 has led me to adhere to the principles of personal hygiene more than before.	7(0.8)	34(4)	54(6.4)	531(62.9)	218(25.8)
15	The outbreak of Covid-19 has reduced unnecessary purchases and increased savings.	45(5.3)	261(30.9)	109(12.9)	349(40.5)	87(10.3)
16	The outbreak of Covid-19has caused me to have fewer accidents due to being more at home.	12(1.4)	91(10.7)	108(12.7)	513(60.5)	124(14.6)
17	The outbreak of Covid-19 has increased my ability to participate in charitable activities in the community (assisting vulnerable individuals).	27(3.2)	187(22.5)	137(16.5)	366(44)	115(13.8)
18	The outbreak of Covid-19 has caused me to do my daily tasks, such as shopping online.	113(13.5)	321(38.4)	113(13.5)	195(23.3)	94(11.2)
19	The outbreak of Covid-19 has made me more confident in the medical staff (doctors and nurses).	18(2.1)	34(4)	82(9.7)	285(33.7)	427(50.5)
20	The outbreak of Covid-19 has led me to pay more attention to the elderly	3(0.4)	27(3.2)	75(8.9)	294(34.9)	444(52.7)

Findings showed that there is a significant association with the variables of age, sex, and occupation status with negative impacts and age, occupational status, history of Covid-19, and family history of Covid-19 with positive impacts (Table 3).

**Table 3:** Comparison of social and demographic characteristics, and mean of positive and negative impacts (*N* = 861)

Variable	Negative Outcome		Positive Outcome	
	Mean (SD)	*P*	Mean (SD)	*P*
Gender				
Male	6.81 (2.17)	*P* = 0.006 t=-2.75	6.68(2.50)	*P* = 0.86 t = 0.17
Female	7.26(2.00)		6.65(2.37)	
Marital status				
Single/divorced	6.71(1.76)	*t* = 2.56 *P* = 0.07	6.87(1.74)	*t* = 9.49 *P* = 0.08
Married	7.21(2.20)		7.23(2.11)	
Work status				
employed	6.98(1.43)	*F* = 3.26 *P* = 0.004	7.68(1.43)	*F* = 2.71 *P* = 0.01
unemployed	6.77(1.76)		6.67(1.76)	
Retired	7.12(1.84)		7.32(1.84)	
Academic student	7.01(1.14)		7.21(1.24)	
housework	7.11(1.26)		6.82(1.36)	
Level of Education				
High school or below	7.26(2.00)	t=-3.421 *P* = 0.13	6.89(2.15)	t= 0.50 *P* = 0.73
College degree	7.56(2.32)		7.12(1.57)	
History of Covid-19				
Yes	6.98(2.41)	t=-1.36 *P* = 0.16	6.39(2.78)	t =-2.3 *P* = 0.01
No	7.20(1.85)		6.80(2.10)	
Family history of Covid-19				
Yes	7.02(2.24)	t=-1.72 *P* = 0.085	6.41(2.60)	t =-3.80 *p* < 0.001
No	7.28(1.75)		7.06(1.86)	
Age	R = 0.085	*P*= 0.01	R = 0.013	*P* < 0.001
Number of children	R = 0.047	*P* = 0.21	R = 0.094	*P* = 0.23

## Discussion

This study aimed to determine the experienced impacts of Covid-19 from a sample of the general population in Tabriz, Iran. In our study, only 61 (7.1%) of individuals mentioned physicians and nurses as the first and second priorities of information sources; for the 736 (85.5%) participants, it was TV/radio, and for 633 (73.3%), it was social networks and internet. Social media is widely available and crucial in transmitting medical knowledge. Information can be constantly updated and disseminated and was vital in the early days of a rapidly evolving outbreak. However, the unregulated nature of the internet can result in unvalidated or unproven information being spread. This can lead to severe and, in some cases, life-threatening consequences [24]. Government and health managers’ attentiveness to these issues and providing the public with increased access to health care providers and valuable information sources could aid in preventing the spread of potentially harmful misinformation.

The findings of the present study showed that the general population experienced many negative impacts of Covid-19, but it is noteworthy that the mean value of positive effects was also high. Literature highlights similar negative findings. In a recent study with a qualitative approach, Italian adolescents reported more negative experiences with Covid-19 [22]. In the United Kingdom, study participants reported higher negative experiences during Covid-19 with limited positive effects of Covid-19 [19]. In Tušl et al., about 30% of employees in Germany and Switzerland reported that their work and private life worsened during Covid-19. Similarly, this study highlighted restrictions on attending cultural and religious sites, gatherings, traveling, and holding mourning ceremonies were the highest frequencies of negative impacts reported by participants [15].

In Iranian culture, religion is a valued component of daily life. A recent Gallup poll reported that 83% of Iranians prioritized religion as a significant part of everyday life [25]. Another national survey focusing on Iranians’ values and attitudes found that over 80% practice prayer regularly as a part of their religion [26]. One might argue that this is a true reflection of an individual culture where prayer and religious beliefs are a part of people’s everyday life. However, for many Iranians, prayer extends beyond solely performing a sacred duty, incorporating different forms of formal and informal practices. Given the limitations that Covid-19 has placed on individuals’ spiritual needs, it is important that healthcare providers be more sensitive to the spiritual dimension of care. Interventions such as enlisting guidance from religious experts when integrating spiritual care with the patients and their families can facilitate meaningful and appropriate counsel.

Covid-19 has also illuminated several positive impacts on people’s lives. Increased adherence to personal hygiene principles, more care for the elderly, more trust in nurses and physicians, fewer accidents due to staying at home, and spending more time with family members were the highest positive effects reported by participants. In the Tusl study, 10% of participants reported improvements in work and 13% in private life [15].

Numerous terms have been used in the literature to describe positive changes in a person related to stress associated with Covid-19. For example, post-traumatic growth and stress-related growth have been used to define an individual transformation that requires positive intrapersonal and interpersonal changes resulting from a difference due to challenges in life [27]. It is suggested that this could be a cognitive strategy used to deflect the harmful effects of traumatic events [28]. Post-traumatic growth enables individuals to reframe their experiences and perceive significant personal growth potential from a major life crisis, which can improve relationships with others, create new possibilities, enhance emotional strength, bring spiritual development, or increase appreciation of life. Growth is not a result of the event itself but rather the struggle to deal with it [29]. In research conducted by Fioretti, adolescents reported increased time of loneliness followed by lockdown and social distancing as a factor in their growth, which leads to more time for thinking [22]. Post-traumatic reconnection with the self includes thinking about the self and accepting situations that cannot be changed. It focuses on the ability to continue living and remember the traumatic incident as a memory from which we learn lessons rather than trying to repress those memories and reject connections with emotions that lead to the development of severe mental health issues [30].

In Iranian religious and national beliefs, respect for the elderly is foundational, and family is considered a sacred unit [31]. In the current study, most participants (87.6%) agreed that the prevalence of Covid-19 has caused them to pay more attention to vulnerable older adults, which can be considered an example of post-traumatic growth. In a research study in Japan, participants identified the positive impacts of Covid-19. They learned how to be kind to others and have a positive and humane attitude toward others [32]. In India, more attention was paid to the care of children and the elderly as one of the effects of Covid-19 [33].

In the present study, promoting healthy behavior and paying more attention to health was scored as one of the most significant positive outcomes of Covid-19. Cleanliness and personal hygiene are essential instructions in Islam traditions [34]. This finding is reinforced by a study conducted by Amirudin, who also highlighted more attention to maintaining cleanliness and regular hand washing as a positive outcome of Covid-19 [21].

In the present study, 81.2% of participants agreed that Covid-19 allowed them to spend more time with family members. The family can play an active role in making sense of life during an epidemic and support individuals when they feel lost [35]. Membership in a family or social/friendship group not only fulfills psychological needs but practical one; family, friends, acquaintances, colleagues, and neighbors constitutes a reliable and permanent support network on which one can call at times of need. The importance of family in Iranian culture and the Islamic religion is so high that Quran considers the family as the source of peace and security [36].

In our study, women perceived more negative impacts resulting from Covid-19. A study in India revealed women having increased pressure during an epidemic due to unbalanced access to economic, social, and health resources [33].

Our study also demonstrated that retired people had higher scores on adverse outcomes. Typically, the retired people in Iran fill their daily time by going to religious and cultural places, ceremonies, parks, and family and friendly parties. Given ongoing restrictions to attending crowded spaces and lack of familiarity with the use of social networks among retired people, this population has experienced more negative impacts, such as sedentary lifestyles and limited social interactions. In comparison, employed people had significantly higher positive impact scores. This can be attributed to the forced shortening of the workday or working remotely, increased leisure time, and time spent with family and friends. Other reasons could be the absence of daily in-person stressors and having more control over their work day [15]. According to Tusl et al., working from home during Covid-19 was associated with higher reports of positive impacts on their personal lives, such as control over the workday, working more efficiently and saving time [15].

This study showed that by increasing age, the participants experienced more (positive or negative) impacts of Covid-19. As people age, they can view aspects with a different lens, and have a more realistic view of life, thus recognizing both the positive and negative factors that might be associated with challenges such as a pandemic. Also, findings showed that women experienced more negative impacts of Covid − 19. In a pandemic, women and girls are most likely to take on extra responsibilities, such as caring for sick persons in the family, losing paid work, and more involvement in distance education, which can affect them negatively [37]. Findings further highlighted that individuals who did not have Covid-19 experiences reported more negative impacts of Covid-19. This finding indicates that individuals who had recovered from Covid-19 may have perceived some positive aspects related to experiencing and surviving the disease.

A strength of the present study is the large and diverse sample size, allowing for a detailed analysis and exploration of different subgroups within the sample. One of the limitations is that the sample population was only residents of Tabriz, Iran, potentially limiting the generalizability to other people.

## Conclusion

The present study’s findings highlighted that the sample population in this study experienced many negative impacts of Covid-19, which may be related to the Iranian culture and religious gatherings of the high importance of Muslims. Additionally, this study revealed positive effects and areas of post-traumatic growth. These findings should not be ignored, and Covid-19 could be used as an opportunity to develop personal growth and a positive outlook on life. More attention should be focused on reinforcing and stabilizing the positive impacts of Covid − 19, such as more personal hygiene and helping vulnerable individuals.

## Data Availability

The datasets generated/analyzed during the current study are not publicly available due to ethical concerns but are available from the corresponding author upon reasonable request. (Ethical committee of Tabriz University of Medical Science has restrictions on the availability of data).
